# Nanostructured Lipid Systems as a Strategy to Improve the *in Vitro* Cytotoxicity of Ruthenium(II) Compounds

**DOI:** 10.3390/molecules19055999

**Published:** 2014-05-09

**Authors:** Eduardo Sinesio de Freitas, Patricia Bento da Silva, Marlus Chorilli, Alzir Azevedo Batista, Érica de Oliveira Lopes, Monize Martins da Silva, Clarice Queico Fujimura Leite, Fernando Rogério Pavan

**Affiliations:** 1 Departamento de Ciências Biológicas, Faculdade de Ciências Farmacêuticas, Universidade Estadual Paulista, Araraquara, São Paulo 14801-902, Brazil; 2 Departamento de Fármacos e Medicamentos, Faculdade de Ciências Farmacêuticas, Universidade Estadual Paulista, Araraquara, São Paulo 14801-902, Brazil; 3 Departamento de Química, Universidade Federal de São Carlos, São Carlos, São Paulo 13565-905, Brazil

**Keywords:** ruthenium complexes, tuberculosis, nanostructured lipid systems

## Abstract

**:** Tuberculosis is an ancient disease that is still present as a global public health problem. Our group has been investigating new molecules with anti-TB activity. In this context, inorganic chemistry has been a quite promising source of such molecules, with excellent results seen with ruthenium compounds. Nanostructured lipid systems may potentiate the action of drugs by reducing the required dosage and side effects and improving the antimicrobial effects. The aim of this study was to develop a nanostructured lipid system and then characterize and apply these encapsulated compounds (**SCARs** **1**, **2** and **4**) with the goal of improving their activity by decreasing the Minimum Inhibitory Concentration (MIC_90_) and reducing the cytotoxicity (IC_50_). The nanostructured system was composed of 10% phase oil (cholesterol), 10% surfactant (soy oleate, soy phosphatidylcholine and Eumulgin^®^) and 80% aqueous phase (phosphate buffer pH = 7.4). Good activity against *Mycobacterium tuberculosis* was maintained after the incorporation of the compounds into the nanostructured lipid system, while the cytotoxicity decreased dramatically, in some cases up to 20 times less toxic than the unencapsulated drug.

## 1. Introduction

Tuberculosis (TB) is an ancient infectious disease whose main causative agent is the bacterium *Mycobacterium tuberculosis*. Despite the technological resources to control the disease, eradication is still a distant goal. The bacterium uses mechanisms that allow it to remain latent for years to evade the host’s immune system. Closely linked to poverty and unequal income distribution, the disease is a serious global public health problem [1].

TB is an ancient disease that is present in society today as never before in history. People who are infected with pulmonary TB can spread the disease by coughing, sneezing or even talking. A patient with pulmonary tuberculosis that is not treated correctly will infect other people, and on average, this individual can transmit the disease to approximately ten to fifteen people per year. The most common symptoms of pulmonary TB are cough, shortness of breath, chest pain, fever, night sweats, loss of appetite, muscle weakness and fatigue [2].

Another factor that contributes to the problem is the fact that approximately one third of the World population is already infected with the bacillus in its latent form. Of these, approximately 10% will develop clinical manifestations, characterized as TB disease, especially individuals co-infected with HIV. Thus, HIV associated with TB has been characterized as a major cause of death among co-infected patients [3].

Modern drug design strategies are based on the knowledge of the pathophysiology of diseases and biochemical pathways for the selection of molecular targets. Modern biotechnological tools have provided valuable information for the discovery and development of new drugs. Medicinal chemistry has a central role in various processes aimed at the identification of bioactive substances and the development of leading-compounds with optimized pharmacodynamic and pharmacokinetic properties [4].

Our group has conducted biological tests to identify bioactive substances from natural products and synthetic organic and inorganic compounds [5,6]. In this approach, our results have shown inorganic chemistry to be a very promising approach. Heteroleptic ruthenium (II) compounds containing phosphines, diimines, picolinates as binders were extremely promising leads, with Minimum Inhibitory Concentrations (MICs) better and/or comparable to those of the current first line drugs [7]. They showed anti-TB activity against both dormant and multidrug-resistant (MDR) bacteria and did not interact with other medicinal treatments already used [8,9].

In recent years, the search for new drug delivery systems has been very relevant in establishing more effective therapeutic alternatives that deliver drugs more safely and with minimized side effects. One of these studies has been directed at microemulsions (MEs), which can be defined as transparent emulsions in which an oil is dispersed in (orderwise) an aqueous medium containing a surfactant, with or without a suitable co-surfactant system generating thermodynamically stable droplets and having an internal phase on the order of nanometers (nm). The active substances can be transmitted when they are solubilized in the MEs in the oily or aqueous phase [10]. The MEs are regarded as reservoir systems because the drug is separated from the dissolution medium through a membrane or interface that must be implemented to allow release into the environment. These systems provide a dimensionally restricted environment with particular properties, such as the ability to bind or associate with molecules of different groups of drugs with the aim of solubilizing, to modulate the amount of drug released, to increase the drug stability in relation to environmental conditions or to improve the profile bioavailability by increasing the concentration of drug at the site of action and preserving the active principle [11].

The formation of microstructures in aqueous surfactant solutions is a common phenomenon of self-organizing molecules as way to achieve stable thermodynamics. This phenomenon is the basis for the technological application of surfactants as organized systems in the biological sciences. Surfactant molecules commonly self-aggregate in the presence of water to form a rich variety of structures whose parameters are varied surfactant concentration, presence of salt and temperature. In dilute solutions, isotropic solutions of micelle aggregates can be formed, while in surfactant-solvent systems at higher concentrations, liquid crystalline isotropic and anisotropic stages can exist. These aggregates become more structured even when an oil or other components such as another surfactant or medium chain alcohol, is added the surfactant-water system. Thus, emulsions, microemulsions and lyotropic mesophases of different geometries can be generated [10].

Importantly, microemulsion systems improve the solubility and stability of drugs, in addition to providing long-acting therapeutics, increasing their bioavailability and decreasing the required dose, targeting specific tissues or organs of the body and delivering active substances with differing degrees of hydrophilicity/lipophilicity in the same formulation [12].

The major focus of this research was the incorporation of ruthenium compounds into microemulsions, to develop an effective therapeutic alternative with lower doses and reduced side effects. Thus, the ruthenium(II) complexes were incorporated into a nanostructured system consisting of soy phosphatidylcholine (SPC) and Eumulgin^®^ (Castor oil polyoxyl-40-Hydrogenated), which are commonly used as surfactants [13,14], sodium oleate (OS) as a co-surfactant [15], cholesterol (CHO) as the oil phase and phosphate buffer pH 7.4. Then, they were evaluated *in vitro* for their antimycobacterial activity against *Mycobacterium tuberculosis* H_37_Rv ATCC-27194 (MIC_90_) and tested on VERO cells (a normal eukaryotic cell) to determine their cytotoxicity (IC_50_).

## 2. Results and Discussion

### 2.1. Nanostructured Lipid System

Light scattering is a routine technique that is used for determining the diameter of the internal phase of microemulsions. Light scattering assays have been developed for liquid microemulsions that have been diluted with deionized water to detect experimental errors [16].

Table 1 shows the mean values and standard deviation of the particle size and polydispersity index for the nanostructured lipid system (ME) and the extracts incorporated in the microemulsion. As per Table 1, the diameter of the ME particles was 129.1 ± 0.9 nm. The incorporation of the complexes caused a small variation in the particle diameter size, without exception, ranging from 167.9 ± 2 to 213.0 ± 3 nm. All values are smaller than 1.0 µm (1000 nm), which is the optimal size for MEs according to Cunha Júnior *et al.* [14]. When comparing the ME and the formulations containing the complexes, there was a small increase in the size of the particle diameter, a strong indication that the complexes were incorporated into the nanostructured lipid system. The polydispersity index was calculated by dividing the mean size of the droplets by the mean number of measured droplets. For both the MEs and the complexes-loaded MEs, the light scattering analysis showed PDI values of 0.144–0.258 indicating a good size distribution of the droplets in the ME system. This parameter directly reflects the size homogeneity of the droplets in the total microemulsion.

**Table 1:** Determination of the droplet size and polydispersity of the ME using light scattering.

Formulation	Mean Diameter ± S.D. (nm) ^*^	Mean PDI ± S.D. ^*^
**ME**	129.1 ± 0.9	0.152 ± 0.01
**SCAR1**	188.0 ± 1	0.211 ± 0.02
**SCAR2**	167.9 ± 2	0.211 ± 0.00
**SCAR4**	213.0 ± 3	0.258 ± 0.01

* Standard deviation (S.D.), polydispersity index (PDI).

### 2.2. Biological Results of Encapsulated and Unencapsulated Ruthenium(II) Complexes

Table 2 shows the MIC_90_, IC_50_ and SI of the compounds. The compounds were tested under two conditions, diluted in DMSO and incorporated into the nanostructured lipid system. According to Pavan *et al.*, 2013 [7,8] and described in the Introduction, the compounds were very active against *M. tuberculosis* pan-susceptible and MDR, as well as latent stage. However, the only study available about the cytotoxicity was published in 2011 and used J774 macrophage cells. In that study, the compounds showed relative cytotoxicity values ranging between 11.90–32.60 µM. In a 2013 study [7] of **SCARs** **2** and **4**, there were five and one mouse deaths, respectively, in the lethal dose (LD_50_) experiment at an oral dose of 2.000 mg/kg/body weight. Based on this study, we decided to study three different complexes containing two ligands (pic and dppb) and changing the nitrogen ligand 2,2'-bipyridine (bipy) found in **SCAR1** with 4,4-dimethyl-2,2'-bipyridine (Me-bipy) in **SCAR2** and with 1,10-phenanthroline (phen) in the **SCAR4**. As shown in Table 2, the compounds solubilized in DMSO are still active against *M. tuberculosis* and relatively toxic as shown in previous work. We also noted that the substitution of the nitrogeneous ligand of **SCAR1** in **SCAR2** and **SCAR4** caused an increase in toxicity. When replacing the bipy ligand with Me-bipy, *i.e.*, substituting two of the hydrogen atoms with two methyl radicals on the pyridine rings, there was a small 2-fold increase, from 47.60 to 22.70 µM, in toxicity; adding an aromatic ring to the nitrogeneous ligand by replacing the bipy with phen in **SCAR4**, also caused a small 1.3-times increase, from 47.60 to 37.60 µM, in the toxicity against VERO epithelial cells. These results indicate that the exchange of only a ligand in the coordination sphere of the Ru(II) compounds most likely alters the mechanism of action, and consequently the toxicity of the resulting complexes.

However, when all compounds were encapsulated, the cytotoxicity decreased drastically and the activity against *M. tuberculosis* was maintained at similar levels as that of the unincorporated compounds. Case by case, **SCAR1** decreased 8-fold, **SCAR2** 22-fold and **SCAR4** 2-fold. These results show that this nanostructured lipid system is able to reduce the cytotoxicity of the complexes; this might be explained by the presence of cholesterol, which could promote interaction with the cellular membrane that consists of a phospholipid bilayer, in the composition of the microemulsion system.

**Table 2:** Results of minimum inhibitory concentration (MIC_90_), cytotoxicity (IC_50_) and selectivity index (SI) of ruthenium(II) complexes alone (unincorporated) diluted in DMSO and incorporated into a nanostructured lipid system (ME).

Identification	Compounds	MIC_90_		IC_50_		SI
		µg/mL	µM	µg/mL	µM	
SCAR 1 (DMSO)	[Ru(pic)(dppb)(bipy)]PF6	1.6	1.7	45.6	47.6	28.0
SCAR2 (DMSO)	[Ru(pic)(dppb)(Me-bipy)]PF6	1.9	1.9	22.3	22.7	12.0
SCAR4 (DMSO)	[Ru(pic)(dppb)(phen)]PF6	1.7	2.0	32.0	37.6	18.8
SCAR1 (ME)	[Ru(pic)(dppb)(bipy)]PF6	6.1	6.4	367.6	383.7	60.0
SCAR2 (ME)	[Ru(pic)(dppb)(Me-bipy)]PF6	2.9	3.0	500	510.2	170.1
SCAR4 (ME)	[Ru(pic)(dppb)(phen)]PF6	2.9	3.4	70.5	82.8	24.4

Considering these two experiments, which involved the substitution of a nitrogeneous ligand in the coordination sphere and the incorporation of coordination compounds into the nanostructured lipid system, we can conclude that the ME is a better transport vehicle than DMSO for these ruthenium complexes.

The selectivity index (SI) of each compound was determined as the ratio of IC_50_ to MIC (Table 2). According to Orme *et al.* [17], candidates for new drugs must have a selectivity index equal to or higher than 10, a MIC lower than 6.25 µg/mL (or the molar equivalent) and a low cytotoxicity. The SI is used to estimate the therapeutic window of a drug and to identify drug candidates for further studies. When we compare the unencapsulated and encapsulated SIs of each compound, we can see a considerable increase. Evaluating each case individually, the selectivity index of SCAR1 increased 2-fold, SCAR2 14-fold, and SCAR4 0.3-fold relative to the unincorporated compounds.

There are studies involving nanoencapsulated drugs as a first line treatment of TB, with the objective of improving the bioavailability of the anti-TB drugs, thereby enhancing the activity of drugs against intracellular *M. tuberculosis* in macrophages and maintaining a therapeutic drug concentration, and consequently improving the compliance of TB patients by developing a new drug delivery system that can be successfully used in interventional technology [18]. However, the association of nanotechnology and inorganic compounds for anti-TB treatment in the literature is very poor, and this is the first time that the ruthenium compounds incorporated into microemulsion are presented as anti-TB drugs. The main goal of this study was to decrease the cytotoxicity and thus reduce the side effects of the medications.

## 3. Experimental

### 3.1. Inorganic Compounds

The synthesis of the complexes [Ru(pic)(dppb)(bipy)]PF6 (**SCAR1**), [Ru(pic)(dppb)(Me-bipy)]PF6 (**SCAR2**), [Ru(pic)(dppb)(phen)]PF6 (**SCAR4**) was performed according to the methodology described by Pavan *et al.*, [8,9].

### 3.2. Nanostructured Lipid System Preparation

MEs were prepared according Formariz *et al.*, [19,20] with modifications and with the following composition: CHO (10%) as the oil phase, PBS (pH 7.4) as the aqueous phase (80%) and a surfactant mixture SPC/SO/EU—3:6:8 (10%). The composition was used to obtain the optimal hydrophilic-lipophilic balance (HLB) value for the stabilization of the clear ME system. The HLB value describes the simultaneous attraction of the surfactant mixture for the oil and aqueous phases; when the HLB is close to the required HLB of the oil phase of the ME, the system provides the minimum energy conditions for ME formation. The composition of the surfactant system SPC/SO/EU was established to obtain an HLB value of 14.97.

The mixture was sonicated using a rod sonicator (Q700 of QSonica^®^, Newtown, CT, USA) at 700 watts in discontinuous mode for 10 min with a 30-second interval in an ice bath every two minutes during the sonication process. After sonication, the MEs were centrifuged at 11,180 *×g* for 15 min to eliminate the waste released by the titanium rod sonicator. MEs were prepared 24 h before the experiments and maintained at 25 ± 0.1 °C to complete the equilibration of the system.

### 3.3. Nanostructured Lipid System Characterization: Mean diameter and Polydispersity Index (PDI)

ME droplet diameters were determined with and without inorganic compounds. All samples were diluted (100 µL sample in 900 µL deionized water). The microemulsion droplet size distribution was determined using dynamic light scattering in a Zetasizer Nano NS (Malvern Instruments, San Diego, CA, USA). The samples were oriented in the analysis chamber so that the laser beam could cross through the dispersion. The temperature of the system was maintained at 20 °C, and the laser wavelength was 532 nm. Ten determinations of the diameter and polydispersity index (PDI) of the drops in each sample were made (*n* = 3).

### 3.4. Preparation of the Coordination Compound-Loaded Nanostructured Lipid System

After obtaining the ME, the coordination compounds were loaded into the nanostructured lipid system. Then, compound (0.0100 g) was added to ME (2 mL) and the mixture was homogenized and sonicated for 5 min at room temperature in discontinuous mode to facilitate the incorporation of the nanostructured material into the lipid system at a concentration of 5,000 µg/mL. The inorganic compound-loaded nanostructured lipid systems were characterized by measuring the mean diameter and polydispersity in a Zetasizer Nano NS (Malvern Instruments, San Diego, CA, USA) and using the Zetasizer Software.

### 3.5. Determination of Minimal Inhibitory Concentration (MIC_90_)

The anti-*M. tuberculosis* activity of the compounds was determined using the Resazurin Microtiter Assay (REMA) method according to Palomino *et al.*, [21]. Stock solutions of the tested compounds were prepared in dimethyl sulfoxide (DMSO) and nanostructured into the lipid systems, then diluted in Middlebrook 7H9 broth (Difco, Detroit, MI, USA) supplemented with oleic acid, albumin, dextrose and catalase (OADC enrichment - BBL/Becton-Dickinson, Detroit, MI, USA) to obtain a final drug concentration range of 0.09–25 µg/mL. A suspension of the *M. tuberculosis* H_37_Rv ATCC 27294 was cultured in Middlebrook 7H9 broth supplemented with OADC and 0.05% Tween 80. The culture was frozen at −80 °C in aliquots. After two days, the CFU/mL of the aliquot was determined. The concentration was adjusted to 5 × 10^5^ CFU/mL, and 100 µL of the inoculum was added to each well of a 96-well microplate together with 100 µL of the compounds. The samples were set up in triplicate. The plate was incubated for 7 days at 37 °C. After 24 h, 30 µL 0.01% resazurin (solubilized in water) was added. The fluorescence of the wells was read after 24 h using a TECAN Spectrafluor^®^ (Männedorf, switzerland). The MIC_90_ was defined as the lowest concentration resulting in 90% inhibition of growth of *M. tuberculosis*.

### 3.6. In Vitro Cytotoxic Activity

The cytotoxicity of the complexes diluted in DMSO and in nanostructured in lipid systems was measured on normal epithelial cells (VERO ATCC CCL -81) as described by Pavan *et al.*, [22]. The cells were incubated at 37 °C with 5% CO_2_ on plates with a surface area of 12.50 cm^2^ in 10 mL DMEM (Vitrocell^®^, Campinas, SP, Brazil) supplemented with 10% fetal bovine serum, gentamicin sulfate (50 mg/L) and amphotericin B (2 mg/L) 

This technique consists of collecting the cells using a solution of trypsin/EDTA (Vitrocell^®^), centrifuging (2000 rpm for 5 min), counting the number of cells in a Newbauer chamber and then adjusting the concentration to 3.4 × 10^5^ cells/mL in DMEM [23]. Next, 200 µL suspension was deposited into each well of a 96-well microplate obtaining a cell concentration of 6.8 × 10^4^ cells/well and incubated at 37 °C in an atmosphere of 5% CO_2_ for 24 h to allow the cells to attach to the plate. Dilutions of the test compounds were prepared to obtain concentrations from 500 to 1.95 µg/mL. The dilutions were added to the cells after the removal of the medium and any cells that did not adhere, and incubated again for 24 h. The cytotoxicity of the compounds was determined by adding 30 µL developer of resazurin and read after a 6-hour incubation. The reading was performed in a microplate Spectrafluor Plus (TECAN^®^) reader using excitation and emission filters at wavelengths of 530 and 590 nm, respectively. The cytotoxicity (IC_50_) was defined as the highest concentration of compound allowing the viability of at least 50% of the cells.

## 4. Conclusions

The ruthenium compounds **SCARs** **1**–**4** have met some of the criteria that a new drug against tuberculosis must fulfill; however, they have considerable cellular toxicity. In this work involving nanoencapsulation, there was a decrease in their cytotoxicity while maintaining their activity against *M. tuberculosis*. These results make this new family of drugs against tuberculosis even more promising.
